# *O*-GlcNAcase: Promiscuous Hexosaminidase or Key Regulator of *O*-GlcNAc Signaling?

**DOI:** 10.1074/jbc.R114.609198

**Published:** 2014-10-21

**Authors:** Jana Alonso, Marianne Schimpl, Daan M. F. van Aalten

**Affiliations:** From the ‡ Medical Research Council Protein Phosphorylation and Ubiquitylation Unit and; § Division of Molecular Microbiology, College of Life Sciences, University of Dundee, DD1 5EH Dundee, Scotland, United Kingdom

**Keywords:** Cell Signaling, Enzyme Inhibitor, Epigenetics, Genetics, Glycobiology, Protein Structure

## Abstract

*O*-GlcNAc signaling is regulated by an opposing pair of enzymes: *O*-GlcNAc transferase installs and *O*-GlcNAcase (OGA) removes the modification from proteins. The dynamics and regulation of this process are only beginning to be understood as the physiological functions of both enzymes are being probed using genetic and pharmacological approaches. This minireview charts the discovery and functional and structural analysis of OGA and summarizes the insights gained from recent studies using OGA inhibition, gene knock-out, and overexpression. We identify several areas of “known unknowns” that would benefit from future research, such as the enigmatic C-terminal domain of OGA.

## Introduction

*O*-Linked *N*-acetylglucosamine (*O*-GlcNAc) is a post-translational modification of serine and threonine residues on nuclear, cytoplasmic, and mitochondrial proteins. Since its discovery in the early 1980s (1), *O*-GlcNAc has been shown to be involved in the regulation of fundamental cellular processes in response to nutritional and hormonal cues (2, 3). *O*-GlcNAcylation has been implicated in gene regulation (4), development (5), signal transduction (6, 7), the cell cycle (8), and proteasomal degradation (9, 10) and shows a degree of interplay with regulatory protein phosphorylation (11). Dysregulation of *O*-GlcNAc levels is associated with a range of human diseases, such as cancer (12), diabetes (13), and neurodegeneration (14).

Protein *O*-GlcNAcylation is a dynamic and reversible process carried out by two single enzymes: *O*-GlcNAc transferase (OGT)^2^ and *O*-GlcNAcase (OGA). Here, we review the literature on OGA, ranging from its discovery 2 decades ago to structural analysis and genetic/pharmacological approaches toward probing its function, revealing several gaps in our knowledge of this key enzyme.

## Discovery and Initial Characterization

**FIGURE 1.**
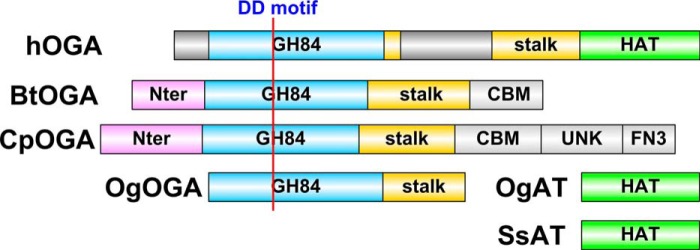
**Schematic of the domain structure of hOGA and apparent bacterial orthologs.** The OGA activity resides in the GH domain (*blue*). The position of the double-aspartate catalytic motif is indicated by the *red line*. The C-terminal domain harbors the putative HAT domain, which is connected via a helical bundle referred to as the stalk domain. Flexible/disordered regions are *gray*: the N-terminal 60 amino acids and the 150-amino acid insertion in the stalk domain. *Cp*OGA, *Bt*OGA, and *Og*OGA have been used to gain insights into the three-dimensional structure of the catalytic domain, whereas putative *Og*AT and *Ss*AT serve as models for the HAT domain. *CBM*, carbohydrate-binding module; *UNK*, unknown module; *FN3*, fibronectin-3 homology module.

The *O*-GlcNAc hydrolase OGA was first purified from rat spleen by Dong and Hart in 1994 (15), a decade after the discovery of the reversible modification of proteins with *O*-GlcNAc. A 1998 study identified it as an antigen expressed by meningiomas and named the gene *MGEA5* (16). The sera of meningioma patients showed immunoreactivity against two apparent alternative splicing isoforms, and initial sequence analyses and biochemical assays suggested hyaluronidase activity (16). However, in 2001, Gao *et al.* (17) performed a thorough biochemical characterization of the recombinantly produced human enzyme, establishing the neutral pH optimum and selectivity for GlcNAc over GalNAc that distinguishes OGA from the lysosomal hexosaminidases HexA and HexB. The existence of an additional hexosaminidase activity (HexC) in mammals had been known since the 1970s (18–20) and was attributed to OGA. Consistent with their subcellular localization, the lysosomal hexosaminidases have an acidic pH optimum, and they cleave terminal *O*-linked β-d-*N*-acetylhexosamine residues irrespective of the C4 configuration (GlcNAc and GalNAc). Their biological role is the degradation of glycans on proteins and lipids, and their dysfunction is associated with lysosomal storage disorders, particularly Tay-Sachs and Sandhoff diseases (21). Despite their distinct physiological functions, the existence of these mechanistically related enzymes pertains to *O*-GlcNAc research, as many frequently used small molecule inhibitors of OGA (most notably PUGNAc) possess little specificity and simultaneously inhibit the lysosomal hexosaminidases. Sequence similarities reveal that OGA belongs to glycoside hydrolase family 84 (GH84) in the CAZy Database (22). A single gene encodes OGA, giving rise to two main isoforms in vertebrates (23) and a single isoform in lower eukaryotes, *e.g. Drosophila* (24) or *Caenorhabditis* (25). The shorter isoform lacks the C-terminal domain but retains some OGA activity (26) and is reported to be localized in the nucleus (27) and in lipid droplets (28). Full-length OGA is a predominantly cytoplasmic and nuclear enzyme (29). In addition to the N-terminal GH84 domain, OGA was observed to possess another domain with potential catalytic activity that belongs to the family of GCN5-related acetyltransferases (Fig. 1) (30). One laboratory has reported that the OGA C terminus possesses histone acetyltransferase (HAT) activity in semipure fractions (27, 31). OGA and HAT activities were speculated to act synergistically, opening up the chromatin structure (acetylation) and activating transcription factors through removal of *O*-GlcNAc (27). However, later studies (32–34) failed to reproduce these observations. Although OGA is post-translationally modified (as reviewed in Ref. 35), the function of these modifications is largely unknown. Similarly, although *O*-GlcNAc levels and OGA/OGT transcription are tightly linked, the mechanisms underlying this are unexplored.

*O*-GlcNAc and OGA have also been described in prokaryotes, and GH84 enzymes are encoded in the genomes of numerous bacterial species. (At the time of writing, there were 185 members of the bacterial branch of GH84 enzymes in the CAZy Database.) Many of these enzymes contain additional domains or secretion signals, leaving their physiological functions somewhat in doubt and wholly unstudied. *In vitro*, however, several bacterial OGAs have been shown to be highly effective in removing *O*-GlcNAc from eukaryotic *O*-GlcNAc proteins and have served as models to study the enzymology of eukaryotic OGAs (36, 37). Intriguingly, a plant ortholog of OGA has not yet been found, and it is not clear whether *O*-GlcNAc in plants is not a dynamic modification or whether convergent evolution has provided an unrelated enzyme that functions analogously to OGA (38).

## Functional Insights from the *O*-GlcNAcase Structure

**FIGURE 2.**
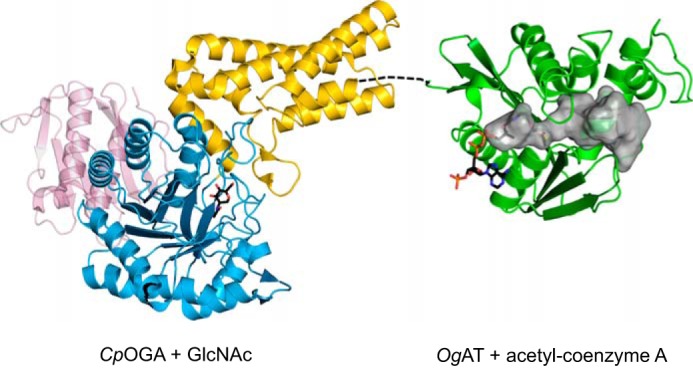
**Crystal structures of *Cp*OGA and *Og*AT serve as models for the human enzyme.** Structures are shown in ribbon representation, with colors as described for Fig. 1. GlcNAc in the active site of *Cp*OGA and acetyl-CoA in the active site of *Og*AT are shown as *sticks* with *black* carbons. The internal surface of the tunnel-like structure in *Og*AT (*gray surface*) may form the binding site for an as yet unidentified ligand.

Human OGA (hOGA) is a 103-kDa enzyme comprising an N-terminal GH84 catalytic domain and a C-terminal HAT-like domain connected by a 300-amino acid region termed the stalk domain (Fig. 1). Although hOGA can be expressed and purified for biochemical studies (29), there is currently no crystal structure for a eukaryotic OGA; however, valuable insights have been obtained from structures of apparent bacterial orthologs (Figs. 1 and 2). The OGA catalytic domain is structurally and mechanistically related to chitinases, *N*-acetylhexosaminidases, and hyaluronidases in the GH18, GH20, and GH56 families (39). These enzymes perform glycoside hydrolysis with net retention of the anomeric configuration via a double-displacement reaction with the carbonyl oxygen of the *N*-acetyl group acting as a nucleophile, a mechanism described as substrate-assisted catalysis or neighboring group participation (40, 41). The active site for the OGA activity of OGA is characterized by a conserved pair of essential aspartic acid residues, Asp-174 and Asp-175, in the human enzyme (41), which constitute the catalytic DD motif. The first insights into the structure of the OGA catalytic domain came from crystal structures of two bacterial OGA orthologs in the GH84 family that were reported in 2006, namely *Clostridium perfringens* NagJ (*Cp*OGA) (36) and *Bacteroides thetaiotaomicron* hexosaminidase (*Bt*OGA) (37) (Fig. 1). The catalytic domain consists of a (β/α)_8_ or TIM (triose-phosphate isomerase) barrel, with the active site located on the C-terminal face of the barrel, where the GlcNAc sugar occupies a deep pocket in the surface of the enzyme (Fig. 2). Both structures were determined in the presence of OGA inhibitors, shedding light on the active site architecture, which then facilitated the targeted generation of catalytically impaired hOGA through the introduction of point mutations. In addition to the D174A/D175A double mutant, which results in loss of catalytic activity, substitution of either aspartic acid with asparagine slows down the reaction and has been employed to trap Michaelis complexes in crystallographic studies (42). The mutation D285A leads to impaired substrate binding. Detailed knowledge of the active site architecture was also exploited for rational design of improved OGA inhibitors, as discussed below. Subsequent work by He *et al.* (42) made elegant use of various substrate analogs to give detailed information about the reaction coordinate of OGA in the form of crystallographic “snapshots.”

Several studies have attempted to address the substrate recognition mechanism of OGA, although it is not yet clear how OGA regulates *O*-GlcNAc levels on hundreds of diverse cellular proteins. Interpretation of the available information is further hampered by the fact that instead of *O*-GlcNAc proteins, most of these studies have relied on the use of glycopeptides or colorigenic/fluorigenic pseudosubstrates. One study observing OGA activity on three different *O*-GlcNAc proteins concluded that the substrate recognition by hOGA occurs in a conserved binding groove and involves the interaction of the enzyme's surface with *O*-GlcNAc proteins (43). This is in apparent contrast to other work showing that β-methyl-GlcNAc, as the “minimal substrate,” was processed more rapidly than a small panel of glycoproteins (44), which led the authors to conclude that any interactions of the protein or peptide with the enzyme are counterproductive. A molecular view of the binding of short *O*-GlcNAc peptides in the active site of a catalytically impaired *Cp*OGA mutant, obtained through crystallographic studies, showed that interactions were limited to the sequence-independent stacking of the peptide backbone to a surface-exposed, conserved, aromatic residue in OGA (45). Conversely, a combination of molecular docking and molecular dynamics simulation suggests that although sequence-independent interactions with the peptide component do contribute, the affinity for OGA substrates varies as a function of the peptide sequence (46). It should be noted that peptide sequence and length may have entropic in addition to enthalpic effects on binding and that their precise conformations in the active site may affect not only binding but also turnover. It is conceivable that substrate recognition does not proceed along the same lines for the entire pool of OGA substrates.

Given that sequence conservation in the bacterial homologs *Cp*OGA and *Bt*OGA is limited to the immediate surroundings of the active site, these were initially thought to provide models for the glycoside hydrolase domain of OGA only. The structure of a third bacterial OGA from *Oceanicola granulosus* (*Og*OGA) was reported in 2010 (43). *Og*OGA shows moderate sequence conservation beyond the catalytic domain, which led to the understanding that the region connecting the N- and C-terminal domains of eukaryotic OGAs comprises a 200-amino acid helical bundle termed the stalk domain and a 150-amino acid unstructured region (Fig. 1). This region harbors Ser-405, the *O*-GlcNAc site on hOGA, which itself is a substrate for OGT (47), as well as residues 404–548, which have been reported to be required for interaction with OGT (48).

The C-terminal domain of OGA is believed to adopt a GCN5 acetyltransferase-like fold (30). Toleman *et al.* (27, 31) reported that semipure fractions of the OGA C terminus showed HAT activity, which has resulted in the domain being referred to as the “putative HAT domain” in the literature, although later studies failed to reproduce these observations. A recent study could not detect binding of acetyl-CoA to the isolated hOGA HAT domain recombinantly expressed in *Escherichia coli* (33). The crystal structure of a protein from the bacterium *O. granulosus* that shows significant sequence identity to the hOGA HAT domain reveals that this domain is likely to be a pseudo-HAT, as its catalytic core appears to lack the key amino acids involved in binding of acetyl-CoA and acetyl transfer onto an acceptor substrate (Fig. 2) (33, 34). Unlike the human protein, *O. granulosus* acetyltransferase (*Og*AT) (33) and a similar protein from *Streptomyces sviceus* (*Ss*AT) (34) readily interact with acetyl-CoA, which is located in a deep substrate-binding pocket in the *Og*AT structure (Figs. 1 and 2). Perhaps most intriguing is the presence of a large tunnel in *Og*AT, conserved in the hOGA HAT domain (as assessed by sequence alignments), which is presumed to be the binding site for an as yet unidentified ligand/acceptor (Fig. 2).

## *O*-GlcNAcase Inhibitors as Tools to Study *O*-GlcNAc Signaling

**FIGURE 3.**
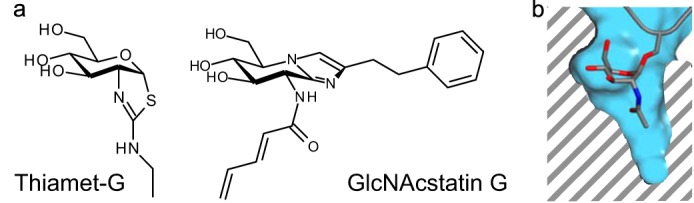
The chemical structures of OGA inhibitors are shown in *a*. Thiamet-G is a representative of the thiazoline compounds, which are stable mimics of the reaction intermediate. GlcNAcstatin G is the most selective member of the GlcNAcstatin family of inhibitors. In both compounds, selectivity has been engineered by elongating the C2 substituent to exploit the depth of the substrate-binding pocket visible in *b*, which shows a cross-section of the active site. GlcNAc is shown in stick representation; the enzyme surface is shown in *blue*.

The elucidation of the OGA catalytic mechanism has enabled the development of potent and selective inhibitors that have subsequently been used to study *O*-GlcNAcylation in cells and organisms. The available inhibitors have been the subject of multiple recent reviews (35, 49–51). Ostrowski and van Aalten (51) focused on their use as probes to study *O*-GlcNAc signaling and covered significant problems with some of the early OGA inhibitors, PUGNAc and streptozotocin, which are unsuitable for cell biological studies due to their off-target effects (35). Here, we will give a brief overview of the two main classes of mechanism-inspired, potent, and selective OGA inhibitors. The catalytic mechanism of *O*-GlcNAc hydrolysis proceeds via an oxazoline reaction intermediate. Stable mimics of reaction intermediates can act as selective enzyme inhibitors, and consequently, thiazoline derivatives of GlcNAc have been explored as OGA inhibitors (Fig. 3). Modifications of the 1,2-dideoxy-2′-methyl-α-d-glucopyranoso[2,1-*d*]-Δ2′-thiazoline (NAG-thiazoline) acyl side chain (39, 52–54) led to improved selectivity over GH18, GH20, and GH56 enzymes that employ the same reaction mechanism. NAG-thiazolines are water-soluble and synthetically accessible OGA inhibitors with potencies in the nanomolar-to-micromolar range, with Thiamet-G being the most selective representative. This family of OGA inhibitors has been described to possess cardioprotective effects: NAG-thiazolines protect against ischemia/reperfusion injury, improving contractile function through preservation of the striated muscle structure (55), and Thiamet-G counteracts TNF-α-induced vascular dysfunction (56). Thiamet-G has also been proposed as a potential therapeutic for Alzheimer disease, as discussed in detail in an accompanying minireview (83). Alzheimer disease and other tauopathies are associated with phosphorylation-dependent oligomerization of tau protein, resulting in neurofibrillary tangles. *O*-GlcNAcylation has been suggested to negatively regulate tau phosphorylation in a site-specific manner (57, 58). Oral administration of Thiamet-G in mice causes an increase in tau S400 *O*-GlcNAcylation and a reduction in neurofibrillary tangles in the brainstem, hypothalamus, and cortex (59). Transgenic mice overexpressing a human tau mutant (JNPL3(P301L)) subjected to long-term administration of Thiamet-G display an increase in motor neurons and body weight paired with a decreased neurogenic atrophy of skeletal muscle (60). Similarly, in a recent study in rTg(tauP301L)4510 mice, Thiamet-G administration caused a remarkable reduction in pathological 64-kDa tau in brain homogenates (61).

The GlcNAcstatin family of inhibitors also owes its design to the elucidation of the catalytic mechanism; these compounds are designed to mimic the transition state (Fig. 3). The most potent GH84 inhibitor designed to date is GlcNAcstatin B, with a *K_i_* of 4.6 pm reported for the bacterial enzyme *Cp*OGA and 0.4 nm for hOGA (62). Selectivity for OGA over the mechanistically related lysosomal hexosaminidases was achieved by extending the *N-*acyl substituent, capitalizing on the presence of a deep pocket in the enzyme's active site that permits OGA to recognize analogs of *O*-GlcNAc (Fig. 3) (63). GlcNAcstatins (the most selective compound being GlcNAcstatin G) are potent cell-permeable inhibitors of hOGA (62, 65) and have been reported to increase *in vivo O*-GlcNAc levels in several human cell lines. GlcNAcstatin G treatment of human embryonic kidney 293 cells overexpressing he IL-1 receptor contributed to the discovery of the role of *O*-GlcNAcylation in activating the TAK1 (TGF-β-activated kinase 1) cascade (66). *O*-GlcNAcylation of the TAK1 accessory protein TAB1, induced by IL-1 or osmotic stress, regulates TAK1 signaling, as *O*-GlcNAcylated Ser-395 on TAB1 is required for full TAK1 activation (66). The use of GlcNAcstatin C (62) to study differentiation of mouse embryonic stem cells revealed that elevated *O*-GlcNAc levels lead to delayed embryonic stem differentiation (67).

## Genetic Approaches toward Probing *O*-GlcNAcase Function

A range of mammalian cell lines have been used to study the possible roles of OGA (68). Contrary to earlier reports, enhanced OGA expression combined with insulin stimulation in 3T3-L1 cells has suggested that a decrease in cellular *O*-GlcNAc does not prevent the onset of insulin resistance (69), also corroborated with studies using selective OGA inhibitors (70, 71). OGA overexpression induces mitotic exit in several cell lines, accompanied by with a delay in mitotic phosphorylation, an altered cyclin expression profile, and a disruption in nuclear organization (72). An involvement of OGA in neuronal development has also been reported in several cell biological studies. OGA overexpression in cultured primary chicken forebrain neurons led to an increase in axon branching (39). Furthermore, OGA transfection prompted neurons to enter neurodevelopmental stage III with an axon with more filopodia (73).

A recent study using orexin neurons (which have a role in circadian rhythm and feeding behavior) has suggested an intriguing link between epigenetics, OGA, and neural differentiation (74). This study showed that OGA and HATs (such as p300 and CBP (cAMP-responsive element-binding protein-binding protein)) are recruited to the transcription initiation site of *Hcrt*, correlating with increased expression of *Hcrt* and H3/H4 hyperacetylation.

A null allele of the gene *oga-1* in *Caenorhabditis elegans* (*ok1207*) results in viable and fertile worms, along with an accumulation of *O*-GlcNAcylated proteins. However, metabolic changes lead to alterations in the insulin-like signaling pathway that controls nutrient storage, life span, and dauer formation (25). Higher levels of *O*-GlcNAc were observed on nuclear pore proteins in *oga-1(ok1207*) animals, and Ser/Thr phosphorylation profiles appeared to be altered. The insulin pathway is linked to life span and stress response, and down-regulation of insulin signaling promotes entry into the dauer stage (75). Consistent with the previous work, Rahman *et al.* (76) discovered that *oga-1(ok1207*) increased the life span of worms by activation of DAF-16, a transcription factor downstream of insulin signaling that induces dauer formation. Finally, the *oga-1* mutant has been proposed as a tool to study neurodegenerative diseases (77, 78). Loss of function of OGA magnifies the severity of neurodegenerative proteotoxicity models in *C. elegans* (MAPT (microtubule-associated protein tau), β-amyloid peptide, and polyglutamine mutant driven to neurons and muscle) (77). The absence of OGA limits autophagosome formation, enhancing the proteotoxicity by the accumulation of toxic protein aggregates (78).

A P-element insertion OGA mutant conferring temperature resistance has been reported in *Drosophila* (79). Tissue-specific knockdown of *oga* in insulin-producing cells or fat bodies resulted in an increase in the production of *Drosophila* insulin-like peptides and an increase in carbohydrate levels in hemolymph, mirroring the insulin resistance phenotype in the *C. elegans oga* mutant (24).

Overexpression of hOGA in zebrafish embryos leads to shortened body axes and reduced brain size, with higher rates of cell death at early developmental stages (80). Epiboly, a key gastrulation movement that plays a significant role in zebrafish morphogenesis (81), is delayed, causing a severe disorganization of the microtubular and actin cytoskeleton (80), proposed to be mediated by *O*-GlcNAc modification of the transcription factor Pou5f1/Oct4 (80).

The first *oga* knock-out mouse model has been recently reported (82). The OGA-deficient mice were generated with a gene-trapped embryonic cell line by inserting the gene trap vector in the first intron of *oga* (64). *oga* disruption leads to neonatal lethality associated with a developmental delay, as well as an increase in *O*-GlcNAc levels in embryos (82). Homozygous *oga*^−/−^ embryos showed a reduction in size and weight, with no visible anatomical abnormalities. At the physiological level, studies of proliferation in *oga*^−/−^ mouse embryonic fibroblasts showed mitotic defects and binucleation, suggesting that cell cycle progression requires a precise control of *O*-GlcNAcylation (82). Mitotic regulators such as aurora kinase B, cyclin B_1_, and Cdc2 are down-regulated in *oga*^−/−^ mouse embryonic fibroblasts (82). It is clear that the results obtained with genetic and pharmacological approaches are not always congruent; this suggests that there are other (noncatalytic) functions of the OGA protein and/or off-target effects of the currently available inhibitors.

## Future Challenges

Although research in the past 2 decades has begun to uncover some of the roles of OGA, suggesting that it is not a promiscuous hexosaminidase but an equal partner of OGT in modulation of the *O*-GlcNAc proteome, many important questions remain unanswered. A single pair of opposing enzymes orchestrates all intracellular *O*-GlcNAc, and ∼1000 proteins have been reported to be *O*-GlcNAc-modified to date. Given the site-specific nature of *O*-GlcNAc and the absence of a strict consensus sequence, the substrate recognition mechanism of the transferase performs a formidable selection procedure. Although it is not clear whether all *O*-GlcNAc proteins are equally subject to deglycosylation by OGA, inhibition of OGA in cells typically leads to a global increase in all detectable *O*-GlcNAc proteins, which is consistent with the interpretation that most *O*-GlcNAc sites on cellular proteins are dynamic and that OGA activity is essential for maintaining normal *O*-GlcNAc levels. Although several studies have addressed OGA substrate recognition, there is no clarity regarding the involvement of the protein part of the substrate. There are no crystal structures of eukaryotic OGAs, let alone complexes with full *O*-GlcNAc proteins, and these will need to be determined to reveal the principles of (differential) substrate recognition.

Although OGA possesses a HAT-like domain, its function is unclear. The OGA isoform that lacks the HAT domain retains some OGA activity, and current data suggest that the hOGA HAT-like domain is not capable of binding an acetyl donor and thus not capable of acetyl transfer. Structural work suggests, however, the presence of an enigmatic tunnel where, in active acetyltransferases, the acceptor substrate normally binds; the potential binding partner for this tunnel remains to be identified. Although there is circumstantial evidence for a role of *O*-GlcNAc and OGA in epigenetics, the possible role of the HAT-like domain will need to be explored.

Although several studies have recently attempted to define the role of OGA by genetic approaches, they have all relied on a total disruption of the gene: abrogating OGA activity but also removing the protein from any complexes of which it might be a component. With the advent of OGA structural data and detailed mechanistic insights, it is now possible to design mutants of the enzyme that are correctly folded yet lack (or are reduced in) catalytic activity. It will be imperative to exploit such mutants in animal models to dissect the catalytic and noncatalytic roles of OGA. We hope that these studies will lead to unification of pharmacological and genetic approaches toward the effects of reducing OGA activity in cell lines and disease models.

## Footnotes

2 The abbreviations used are:

OGT: *O*-GlcNAc transferase
OGA: *O*-GlcNAcase
hOGA: human OGA
*Cp*OGA: *C. perfringens* OGA
*Bt*OGA: *B. thetaiotaomicron* OGA
*Og*OGA: *O. granulosus* OGA
GH84: glycoside hydrolase family 84
HAT: histone acetyltransferase
*Og*AT: *O. granulosus* acetyltransferase
PUGNAc: *O*-(2-acetamido-2-deoxy-d-glucopyranosylidene)amino *N*-phenylcarbamate
*Ss*AT: *S. sviceus* acetyltransferase
NAG-thiazoline: 1,2-dideoxy-2′-methyl-α-d-glucopyranoso[2,1-*d*]-Δ2′-thiazoline.
